# Assessment of undernutrition using the composite index of anthropometric failure (CIAF) and its determinants: A cross-sectional study in the rural area of the Bogor District in Indonesia

**DOI:** 10.1186/s40795-022-00627-3

**Published:** 2022-11-16

**Authors:** Tria Astika Endah Permatasari, Yudi Chadirin

**Affiliations:** 1 Department of Nutrition, Faculty of Medicine and Health, Universitas Muhammadiyah Jakarta, Central Jakarta, 10510 Indonesia; 2 Department of Civil and Environmental Engineering, IPB University, Dramaga, West Java 16680 Indonesia

**Keywords:** Composite Index of Anthropometric Failure (CIAF), Stunting, Undernutrition, Underweight, Wasting

## Abstract

**Background:**

The Composite Index of Anthropometric Failure (CIAF) can comprehensively identify undernutrition by combining several indicators of nutritional status – namely, weight-for-age, length/height-for-age and weight-for-length/height – to determine the nutritional status of children under five years of age. This study aims to assess undernutrition using the CIAF and its determinants on children under five years of age in the Bogor District, Indonesia.

**Methods:**

A cross-sectional study was conducted during February–May 2019 among 330 mother-children pairs (with children under five), selected by systematic random sampling from four villages as undernutrition pockets in the rural area of Bogor District, Indonesia. The nutritional status of the children was assessed by measuring weight and length/height. Z-score was calculated using WHO Anthro software and was categorized based on conventional indices, including weight-for-age (WAZ), length/height-for-age (HAZ) and weight-for-length/height (WHZ). The CIAF is measured based on a combination of conventional index measurements. In addition, mothers’ and childrens’ characteristics and clean living behaviour are assessed via structured questionnaires. Environmental sanitation is assessed using the environment meter. Binary logistic regression analysis with SPSS version 22.0 is used to analyse the dominant factors associated with undernutrition.

**Results:**

Among children under five, 42.1% experienced anthropometric failure (overall prevalence of undernutrition based on the CIAF), 2.4% experienced wasting only, 5.8% were classified as both wasting and underweight, 2.1% as wasting, underweight and stunting, 16.4% as underweight and stunting, 11.5% as stunting only, and 3.9% as underweight only. Assessment of nutritional status using a conventional anthropometric index shows that respective prevalences of underweight, stunted and wasted were 27.8, 29.7, and 10.6%. The mother’s height is the most dominant factor associated with anthropometric failure [*p* = 0.008; AOR = 1.95; 95% CI: 2.19–3.19]. The most dominant factors associated with the conventional undernutrition indices of underweight, stunted and wasted are, respectively, family income [*p* = 0.018; AOR = 5.44; 95% CI: 1.34–22.11], mother’s height [*p* = < 0.001; AOR = 3.29; 95% CI:1.83–5.91] and child’s age [*p* = 0.013; AOR = 2.59; 95% CI: 1.22–5.47].

**Conclusion:**

Nearly half of children under five experience anthropometric failure. Specific nutrition improvement interventions and specific nutrition interventions during pregnancy and lactation are needed, especially for malnourished mothers, to prevent malnutrition in infant.

**Supplementary Information:**

The online version contains supplementary material available at 10.1186/s40795-022-00627-3.

## Background

Undernutrition in children under five years of age is a problem in many countries. In Asia, 49.9% of children under five experience growth failure (i.e. are stunted, wasted or overweight) [1]. Likewise in Indonesia, as one of the countries in Southeast Asia, there is a high prevalence of stunting. In 2018, the prevalence of stunting in Indonesia (30.8%) was the second-highest in Southeast Asia, after that of Cambodia [2]. This figure signifies a challenge for the Indonesian government, which is aiming for a 14% reduction in the prevalence of stunting by 2024 [3]. Based on the results of the 2018 Basic Health Research (Riskesdas), the prevalence of stunting in Indonesia showed a decline of 6.4% over five years, from 37.2% (2013) to 30.8% (2018). However, due to some other nutritional problems, as many as 17.7% were underweight and 10.2% were wasting. This malnutrition problem occurs evenly across various provinces. In West Java Province, there is 31.1% stunting, 13.2% underweight and 8.4% wasting. This serious problem can affect a child’s health, making them more susceptible to disease and infection, and impairing their mental and physical development [4, 5]. Furthermore, this nutritional problem can hinder economic growth and reduce labor market productivity, contributing to widening inequality and causing poverty across generations [6, 7].

Efforts to deal with nutritional problems in Indonesia have been inititated since the early 1980s, through observation at the village level using integrated weighing and child health in integrated post services (Posyandu) [8]. This commitment is strengthened through Presidential Regulation number 72 of 2021, which concerns the Acceleration of Stunting Reduction [3]. This effort is in line with one of the 2030 Sustainable Development Goals (SDGs), namely, eliminating all forms of malnutrition, including reducing the prevalence of stunting and wasting for those under the age of five by 2025. Undernutrition is a global problem: One in three children under the age of five suffers from stunting, wasting, overweight and in some cases suffer a combination of the two forms of malnutrition [1].

In the population, indicators of undernutrition in children can be identified by anthropometric measurements. This method is used to assess the size, proportion and composition of the human body. Anthropometry can be used to evaluate the general health status, nutritional adequacy and growth and development patterns of children [9, 10]. The World Health Organization (WHO) report presents the first set of WHO Child Growth Standards using the conventional indices of weight-for-age, length/height-for-age, weight-for-length/height and BMI-for-age [11, 12]. In Indonesia, assessing a child’s nutritional status is stipulated in Regulation of the Minister of Health of the Republic of Indonesia Number 2 of 2020 concerning Child Anthropometry Standards. There are four conventional indices: weight-for-age, height/length-for-age, weight-for-height/length (for children aged 0–60 months) and BMI-for-age (for children aged 0–60 months and children aged 5–18 years) [13].

The weight-for-age index indicates general nutritional problems and represents a child’s current nutritional status. This index is used to assess children who are underweight or severely underweight. The height/length-for-age index indicates chronic malnutrition. This index can identify children who have suffered from frequent illness or malnutrition for a long time; it is categorized into short (stunted) or very short (severely stunted). This nutritional problem is associated with high poverty, unhealthy living behaviour, parenting patterns, failure to give exclusive breastfeeding, early breastfeeding, complementary foods and low balanced nutrition practices [14–16]. It is also related to infectious diseases such as diarrhea, Acute Respiratory Infections (ARI) and tuberculosis [14, 17–19]. The high prevalence of infectious diseases is also poor environmental sanitation and lack of hygiene practices [20]. The weight-for-height/length index describes a recent (acute) or prolonged (chronic) condition of poor nutrition. It is categorized into undernutrition (wasted), severe undernutrition (severely wasted) and possible risk of overweight. BMI-for-age is used for assessments in accord with the categories of poor nutrition, poor nutrition, good nutrition, risk of overnutrition and obesity. This index is more sensitive when screening for overweight and obese children. However, the four conventional indices cannot determine the overall prevalence of malnutrition in the population. Researchers are forced to choose one category of anthropometric failure to represent the nutritional status of the target population while foregoing information on the other nutritional indices [13].

The Composite Index of Anthropometric Failure (CIAF) method was developed to overcome multiple nutritional failures and to report the prevalence of accurate data. This method identifies children with single or multiple anthropometric failures [21]. The CIAF is an anthropometric index that combines the three indices of weight-for-age, height/length-for-age and weight-for-height/length, so as to determine the nutritional status of children under five years of age [22]. The combined index method of the Svedberg model divides malnourished children into six categories: A) without anthropometric failure; B) wasting only; C) wasting and underweight; D) wasting, underweight and stunting; E) underweight and stunting; F) stunting only. Furthermore, Nandy et al. added category Y), which is underweight only [23]. The CIAF is right in emphasising the importance of child feeding practices, family planning practices, appropriate mothers’ parenting patterns and mothers’ knowledge in preventing the prevalence of undernutrition in children under five years. This measurement model can accelerate the reduction in child mortality by expanding preventive and curative interventions that are more effective in addressing the significant causes of undernutrition. The CIAF also describes a comprehensive measure and can detect children with multiple anthropometric failures [24]. In light of this, Svedberg added three categories to the existing conventional indicators: C) wasting and underweight; D) wasting, underweight and stunting; E) underweight and stunting [25].

In Indonesia, the CIAF is frequently applied to identify nutritional problems; the results show that the prevalence of under-fives who experience anthropometric failure is still high [26, 27]. Until now, however, the measurement of the nutritional status of children under five at the national level has still used the conventional anthropometric index, which only assesses one type of undernutrition. The results of measuring nutritional status using the conventional index are still high. If the measurement of nutritional status only uses one indicator of nutritional status, it is possible to lose information on other malnutrition problems. This will affect the response effort, because the reference information used does not represent the actual problem. That is why the prevalence of undernutrition is still quite high; efforts to tackle nutrition problems have not been comprehensively carried out. Therefore, the measurement of nutritional status using the CIAF constitutes a solution, as it enables the prevention of undernutrition to be more appropriately based on the type of undernutrition experienced by children under five [28].

Previous studies have shown that the prevalence of undernutrition in children under five years is still high. Porwal et al. (2021) reported that, in India, 48.2% of children experienced anthropometric failure. The causes of this anthropometric failure are low household income and residence in urban areas. Moreover, according to the CIAF, a high risk obtains for children of mothers who are underweight and have many children [29]. A similar study in Ethiopia on anthropometric measurements using the CIAF method showed that the child’s age, previous birth spacing, mother’s educational status, wealth status and region were independent factors related to the nutritional status of children in rural Ethiopia [30]. Another study showed that the risk of anthropometric failure was higher among older children who had low birth weight, had mothers with low BMI, resided in rural areas, had mothers and fathers without formal education [31]. A study conducted by Das et al. in Bangladesh also reported that differences in demographic characteristics such as age, gender and type of residence were significantly related to the occurrence of various types of undernutrition including wasting, underweight and stunting, with the prevalence of all forms of undernutrition showing an increasing trend in both rural and urban areas [32]. In addition, the occurrence of various types of undernutrition is also associated with the impact of environmental exposure. Poor environmental sanitation conditions and unfavorable temperature exposure can increase the risk of undernutrition in children under five [33]. The availability of clean water for drinking and for household needs, as well as sanitation and hygiene conditions, are positively related to the occurrence of undernutrition and infectious diseases of the digestive tract [34]. Therefore, the assessment of undernutrition using the CIAF is necessary for overcoming the problem of malnutrition, especially in Indonesia.

## Methods

### Study area and period

Bogor Regency is a buffer zone for the national capital, namely, DKI Jakarta Province. According to data from the Central Statistics Agency (CSA), the population in Bogor Regency was 5,427,068 people in 2019, the highest in the West Java Province. Bogor Regency is not only the district with the most population in West Java Province; it also occupies the first rank of the district with the most population in Indonesia. In 2019, the population growth rate of Bogor Regency was 2.13%, but during the COVID-19 pandemic, the population in this region decreased by around 500 thousand people. The population in Bogor Regency shows a pyramid of youth. Of the total population in this region, as many as 9.68% were children aged 0–4 years in 2019. This number decreased from 2016 to 2019. Conversely, the infant mortality rate and toddler mortality rate in Bogor Regency is high [35].

Bogor Regency is included in the 100 priority areas for stunting interventions in Indonesia [6]. The prevalence of stunted children in this region reached 32.9% in 2018; this number is included in the category of high prevalence, based on the WHO category (30–39%) [36]. Likewise, malnutrition and underweight are still the focus of serious handling in this region. This district has 101 health centres spread over 40 sub-districts, with several sub-districts being included in the category of nutrient-prone areas. Sub-districts located in the lowlands, namely, in urban areas, have developed into industrial areas. Meanwhile, the sub-districts in the highlands have been developed as agricultural areas. Resident alternatives to obtain quality drinking water sources vary widely. Most urban communities have used the services of regional drinking water companies to meet their needs for drinking water sources. In rural areas, people’s options are more varied, ranging from protected wells, dug wells, hand pumping wells, protected springs, rainwater reservoirs to water bodies such as lakes and rivers, as means for meeting their drinking water needs. The population in general work as factory workers, farmers, entrepreneurs and a small part as government employees. More than half of the population has low education. Women tend to have less education than men. They generally marry before the age of 18, becoming housewives and acting as the primary caregivers for their children [37].

### Design and samples

A cross-sectional study was conducted from February to May of 2019 in rural areas of Bogor District, West Java Province, Indonesia. A total of 330 pairs of mothers and children under five participated in this study. Participants came from two community health centre working areas in one sub-district, a nutrient-prone area in Bogor Regency. Four out of 10 villages that became pockets of undernutrition were selected as locations for this study. Participants who met the inclusion criteria and exclusion criteria were selected from the four villages by systematic random sampling.

### Inclusion and exclusion criteria

Participants in this study are mothers with children aged 0–59 months who have lived for at least six months or more in the village. Thus, the study could obtain homogeneous exposure to people’s lifestyles, as well as access to information and health services in rural areas. Other inclusion criteria were that the children were not undergoing intensive health care or suffering from serious illnesses that affect their nutritional status. The exclusion criterion ruled out children who had congenital abnormalities from birth.

Participants for this study were recruited through the following steps. First, Bogor District Health Office reported two working areas of community health centres in one sub-district with the highest prevalence of undernutrition. From each community health centre, two villages were selected which were undernutrition pockets (a total of four villages which are undernutrition pockets). At the first community health centres, there were 268 eligible mother-child pairs, while for the second community health centres there were 270 eligible mother-child pairs. A number of mother-child pairs were excluded, and some were migrants who had lived in the area for less than six months. Therefore, as many as 253 mother-child pairs obtained for the two villages in the first community health centres, and 254 mother-child pairs for the two villages in the second community health centres, met the inclusion and exclusion criteria. From these, participants were recruited using a systematic random sampling technique, whereby participants were selected systematically by using a certain interval (distance) from a sample frame that had been sorted. The sample frame was obtained from data from each community health centre. In order to meet the number of sample requirements, a total of 330 mother-child pairs were selected from two community health centres (four villages); that is, 165 mother-child pairs from each community health centre.

### Sample size determination

The sample was calculated using the one–sample test of proportions with a two-sided alternative hypothesis, with the following assumptions: 5% level of significance, 90% power, 48.5% undernutrition among rural children (*P*_*0*_) based on a previous study [30], and *P*_*a*_ 10% smaller than *P*_*0,*_ and 10% contingency for loss to follow-up. Therefore, the calculated sample size was 330 mother-child pairs [38].

1 $$\boldsymbol{n}=\frac{{\left\{{\boldsymbol{Z}}_{\textbf{1}-\boldsymbol{\alpha} /\textbf{2}}\sqrt{{\boldsymbol{P}}_{\textbf{0}}\left(\textbf{1}-{\boldsymbol{P}}_{\textbf{0}}\right)}+{\boldsymbol{Z}}_{\textbf{1}-\boldsymbol{\beta}}\sqrt{{\boldsymbol{P}}_{\boldsymbol{a}}\left(\textbf{1}-{\boldsymbol{P}}_{\boldsymbol{a}}\right)}\right\}}^{\textbf{2}}}{{\left({\boldsymbol{P}}_{\boldsymbol{a}}-{\boldsymbol{P}}_{\textbf{0}}\right)}^{\textbf{2}}}$$

### Sampling technique and procedure

**Fig. 1:**
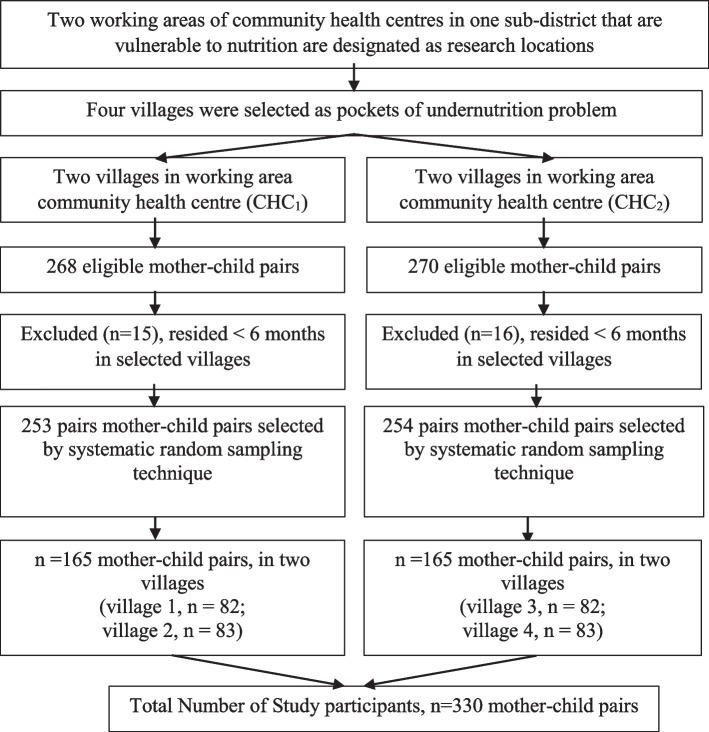
Sampling procedure of mother-child pairs

Four villages as pockets of undernutrition were selected from two working areas of the community health centre in the Bogor District. Eligible participants were selected using a systematic random sampling technique in illustrated Fig. 1.

### Data collection and measurements

#### Anthropometric measurements

Anthropometric measurements were performed using conventional indices, namely, weight-for-age, length/height-for-age and weight-for-length/height. In addition, measurements applied the CIAF, which is an anthropometric index that combines the three indices of weight-for-age, length/height-for-age and weight-for-length/height to determine the nutritional status of children under five years. Age was obtained from information provided by the mother, namely, the date, month and year the child was born. Then the child’s age is calculated in months. The anthropometric measurement technique used in this study was carried out in accordance with the WHO Child Growth Assessment protocol and measurement standards [12].

#### Weight-for-age

The anthropometric measurements using weight-for-age were carried out to assess underweight children. For children less than two years of age, bodyweight was measured using the SECA 725 digital infant scale. This scale can weigh babies up to 16 kg with a sensitivity level of 5 g. Babies are weighed using light clothing and without diapers. Meanwhile, for children older than 24 months, a SECA 876 digital floor scale with a measuring capacity up to 250 kg (accuracy 0.01 kg) was used. Before being weighed, footwear and accessories such as hats, watches and other objects were removed; the clothes used were not thick. Weight measurement was carried out twice, and the results were averaged. The distance between measurements was not more than 0.02 kg. The scale was calibrated prior to use. Furthermore, the categorization of nutritional status is the child’s growth standards according to WHO, which is analysed with WHO Anthro software [12]. Based on the z-score, nutritional status was categorized as one of the following:severely underweight (< − 3 SD); underweight (− 3 SD to < − 2 SD); normal weight (− 2 SD to + 1 SD); risk of overweight (> + 1 SD) [11, 12]. Based on on the explanation contained in Regulation of the Minister of Health of the Republic of Indonesia Number 2 of 2020 about Child Anthropometry Standards, Child Anthropometry Standards in Indonesia refer to WHO Child Growth Standards for children aged 0–5 years and the WHO Reference 2007 for children 5–18 years of age. These standards show how a child’s growth can be achieved if certain conditions are met. Research shows that children from any country will grow up similarly if proper nutrition, health and parenting are in place. As a result of various studies and expert discussions, Indonesia decided to adopt this standard as an official standard to be used as an anthropometric standard for assessing children’s nutritional status, through the Decree of the Minister of Health of Indonesia Number 1995/Menkes/SK/XII/2010 concerning Anthropometric Standards for Assessment of Child Nutritional Status [13].

#### Length/height-for-age

The anthropometric measurement using the length/height-for-age index was applied to assess stunted children. For children under two years old or unable to stand, length is measured using a SECA 210 length board with a measuring range of 10–99 cm and graduation (measuring rod) of 5 mm; the device is placed on a table or flat surface; then the baby is laid on their back without a hat/headcover or footwear. The baby’s feet must be close together; the baby’s knees are pressed until they are straight, and the soles of the feet are straightened. Meanwhile, for children who are more than two years old or who can stand up, height is measured by a GEA stature meter (measuring up to 2 m). The child should stand straight with relaxed arms facing the thighs. The top part of the stadiometer is pressed to the crown of the head so that it sticks to the horizontal part of the stadiometer. The buttocks, shoulder blades and heels attach to the back of the stadiometer. In addition, the child’s feet form an angle of 60 degrees and face outwards. The length/height measurement was conducted twice; then the results were averaged. The distance between measurements was not more than 0.2 cm. All length/height measuring instruments used have an accuracy of 0.1 cm, and the tools were calibrated before use. The results are analysed by WHO Anthro software. Nutritional status categorization is the child’s growth standards according to WHO (World Health Organization). Based on the z-score, nutritional status was categorized into the following: severely stunted (< − 3 SD); stunted (− 3 SD to < − 2 SD); normal − 2 SD to + 3 SD; tall (> + 3 SD)) [11, 12].

#### Weight-for-length/height

The weight-for-length/height index is a good indicator for assessing the current nutritional status of children under five. This index is independent of age, so it does not require data on the ages of the children. The weight gain will be in line with the height at a certain speed. The practice of measuring weight and height in toddlers in assessing nutritional status with an index applies the same principles as anthropometric measurements for the indices of weight-for-age and length/height-for-age. After being analysed with the WHO Anthro software, the nutritional status is categorized based on the z-score into six categories: 1) severely wasted (< − 3 SD); 2) wasted (− 3 SD to < − 2 SD); 3) normal (− 2 SD to + 1 SD); 4) possible risk of overweight (> + 1 SD to + 2 SD); 5) overweight (> + 2 SD to + 3 SD); 6) obese (> + 3 SD) [11, 12].

#### Composite index of anthropometric failure (CIAF)

The CIAF is a concept introduced by Svedberg. It is an alternative index for measuring the failure of nutritional status, which is then accumulated into one index value that can represent the prevalence of malnutrition as a whole [39]. Measurement of nutritional status using one single index renders it difficult to distinguish the occurrence of combined forms of undernutrition, because there is overlap – so it cannot reflect the anthropometric failure rate in a population. The CIAF measurement is based on the Handbook of Anthropometry: Physical Measures of Human Form in Health and Disease [21]. The CIAF is an anthropometric index that combines the three indices of weight-for-age (WAZ), length/height-for-age (HAZ) and weight-for-length/height (WHZ) to determine the nutritional status of children under five. The category of undernutrition based on the CIAF is divided into anthropometric failure and no failure (normal) (Table 1). The categories are grouped into: A) no failure (normal); B) wasting only; C) wasting and underweight; D) wasting, underweight and stunting; E) underweight and stunting; F) stunting only; Y) underweight only. The anthropometric failure is the total amount of undernutition or sum of category of wasting only (B), wasting and underweight (C), wasting, underweight and stunting (D), underweight and stunting (E), stunting only (F) and underweight only (Y). At the same time, the CIAF index can be used to detect some anthropometric failures [39].

**Table 1:** Category Anthropometric Failure in under five year using Composite Index of Anthropometric (CIAF)

Group	CIAF Categories	Description of the level	Wasting	Stunting	Underweight
A	No failure	Normal WAZ, HAZ and WHZ	No	No	No
B	Wasting only	WAZ < − 2SD, but normal HAZ and WHZ	Yes	No	No
C	Wasting and underweight	WAZ and WHZ < − 2 SD, but HAZ normal	Yes	No	Yes
D	Wasting, underweight and stunting	WAZ, WHZ and HAZ < − 2SD	Yes	Yes	Yes
E	Stunting and undwerwight	HAZ and WHZ < − 2SD, but WAZ normal	No	Yes	Yes
F	Stunting only	HAZ < −2SD, but normal WAZ and WHZ	No	Yes	No
Y	Underweight only	WHZ < −2SD, but normal HAZ and WA	No	No	Yes

Source: The Concept of Composite Index of Anthropometric Failure (CIAF) by Kuiti and Bose: Revisited and Revised (2018)

#### Mother and child characteristics

The questionnaire used to assess the characteristics of mothers and children in this study was prepared in reference to the 2018 Basic Health Research questionnaire developed by the Ministry of Health of the Republic of Indonesia, as this questionnaire can be used in general by the public without a license (open access) [40]. Mother and child characteristics were measured by direct interviews, using a structured questionnaire. Mother’s age is categorized based on the mean age (25 years) of the mother, i.e. 1) mother’s age is below 25 years or 2) mother’s age is equal or more than 25 years. Mother’s height is categorized based on the mean value, i.e. 1) short, if the height is less than 150 cm, and 2) tall, if more than or equal to 150 cm. Mother’s education is categorized as 1) low, for those who never attended school and have a primary school education, or 2) high, for those who have at least a high school education. Mother’s employment status is grouped into 1) not working and 2) working.

The level of family income is the amount of income received by the family per month. It is then grouped based on the regional minimum wage in Bogor District (IDR 3.800.000), that is 1) low income, if less than the regional minimum wage, or 2) high income, if equal to or greater than the regional minimum wage. Parity is the number of children born to the mother, grouped into 1) primipara, if the mother has given birth to only one child, 2) multipara, if the mother has given birth to 2–4 children, and 3) grand multipara, if the mother has given birth to more than four children. The primary caregiver is the person who takes care of the children on a day-to-day basis. These variables are categorized into 1) being raised by someone other than the mother and 2) mother as a primary caregiver.

The mother’s nutrition knowledge questionnaire has been developed in this study and used, having been shown to be valid and reliable. Data regarding the mother’s knowledge was measured by asking 20 questions about balanced nutrition and undernutrition of children (Supplementary File 1). Mothers were asked to answer with a choice of “true” or “false” for each statement. Each correct answer was given a score of 1, and each incorrect answer was given a score of 0. The total score obtained is divided by the number of questions multiplied by 100% (correct answer score/20 × 100%). Based on the normality test using the Kolmogorov Smirnov test, the nutrition knowledge score data was normally distributed, so that the mean value was 70. The mean value is also used as a cut-off point in categorizing maternal nutritional knowledge, so that it provides a picture of maternal nutritional knowledge based on population characteristics, namely, in rural areas. Furthermore, mother’s knowledge is categorized into 1) not good, if the mother was able to answer less than 70% of the nutrition knowledge questions correctly, and 2) good, if the mother was able to answer at least 70% of the nutrition knowledge questions correctly.

Characteristics of children (sex, age, immunization history and early initiation of breastfeeding) were measured using a structured questionnaire. The sex of a child is categorized into 1) boy or 2) girl. A child’s age is categorized into 1) below 24 months or 2) equal or more than 24 months. Immunization history is the provision of complete basic immunizations that must be given to infants from birth to nine months of age and categorized into 1) ungiven, if the baby from the beginning of birth to the age of nine months was not given any immunizations at all, 2) incomplete, if the baby had not been fully immunized and was not nine months old, or 3) complete, if the baby was given all types of immunization from birth to nine months of age. Early Initiation of Breastfeeding (EIB) is categorized into 1) No, if the baby was not given breast milk immediately after birth for the first hour, or 2) Yes, if the baby was breastfed immediately after birth until the first hour after birth.

Data regarding the frequency of consumption of food sources of energy and the frequency of consumption of food sources of protein in children were measured by the Food Frequency Questionnaire (FFQ). In this study, the FFQ was compiled by reference to the Indonesian version developed by the Ministry of Health of the Republic of Indonesia used to conduct food consumption surveys. The original questionnaire can be accessed widely (open access). The list of names contained in the FFQ has been grouped into food ingredient groups, such as staple food, animal side dishes and vegetable side dishes. The types of food available in the FFQ have been adjusted to the types of food commonly consumed in Indonesia [41]. Systematic measurement of the FFQ method begins with a preliminary study, which aims to identify food ingredients to be included in the FFQ list. The list of foodstuffs is adjusted according to the magnitude of the correlation with the risk of exposure to consumption and the occurrence of undernutrition in children under five, as well as the availability of food to the population and a high frequency of consumption [42, 43].

The FFQ form’s structure consists of three main columns: 1) number; 2) food and beverage ingredients; and 3) frequency of consumption. The frequency of eating column is divided into six sections, each of which is a selection of eating frequency items. The choice of eating frequency items is divided according to the frequency of consumption, as follows: 1) more than three times a day; 2) once a day; 3) three to six times a week; 4) one to two times a week; 5) twice a month; 6) never. After receiving instructions for filling out the FFQ, each mother filled out the form herself. Each mother gives a checklist on the frequency of food consumption for each type of food listed in the FFQ consumed by her child in the last three months. For food consumption of energy sources, based on the analysis, results are categorized as 1) low consumption, if the consumption frequency is below three times a day (< 3x/day), or 2) high consumption, if the consumption frequency is equal to or more than three times a day (≥ 3x/day). Protein source food consumption is categorized into 1) low consumption, if the protein consumption frequency is below three times a day (< 3x/day), or 2) high consumption, if the protein consumption frequency is equal or more than three times a day (≥ 3x/day).

#### Environment sanitation and clean living behaviour

Measurements of clean living behaviour include sources of drinking water, hand-washing habits and bowel habits, while measurements of environmental sanitation include indoor air temperature and relative humidity. Measurements of clean living behaviour were taken using a questionnaire based on the 2018 Basic Health Research questionnaire developed by the Ministry of Health of the Republic of Indonesia (open access questionnaire) [40]. Environmental sanitation measurements were not measured for every house, because not every participant allowed data collectors to enter the house. Some participants explained that the condition of their house was not suitable and stated that they were disturbed.

Measurements of ambient room temperature were conducted using an environmental meter (Krisbow KW0600291). Ambient room temperature was measured by placing the instrument at chest level, waiting for five minutes, to measure the temperature steadily. The measurement protocol refers to SNI 16–7061-2004 (Measurement of Work Climate). It was measured twice, and the results were recorded and averaged. The measurement results were then categorized based on SNI T-14-1993-037 about the Thermal Comfort Zone Standard in Indonesia (based on effective temperature) into: 1) uncomfortable, if the ambient room temperature was below 20.5 °C or more than 27.2 °C, or 2) comfortable, if the ambient room temperature ranged between 20.5–27.2 °C.

The home’s relative humidity (RH) is measured by the environmental meter (Krisbow KW0600291). This tool can measure the air’s relative humidity in the range of 35%RH ~ 95%, resolution 0.1%RH, with an accuracy level (%rdg + digits) of ±5%RH at 25 °C. Measurement of relative humidity of the air was conducted by placing the instrument at chest level, waiting for five minutes, to measure the temperature steadily. The measurement protocol is based on SNI 16–7061-2004 (Measurement of Work Climate). It was measured twice, and the results were averaged and recorded. These results were then categorized based on SNI 03–6572-2001 about Standards, Procedures for Designing Ventilation and Air Conditioning Systems in Buildings into 1) uncomfortable, if the relative humidity was below 40% or more than 70%, or 2) comfortable, if the relative humidity ranged between 40 and 70%.

Sources of cooking water were categorized into 1) unprotected springs and 2) protected springs. Handwashing habits are divided into two categories, 1) not good, if washing hands did not involve running water and soap, 2) good, if washing hands does involve the use of running water and soap. Also, the habit of defecating is divided into 1) open place, if defecation is carried out in open places, such as rivers and gardens, or 2) toilet.

#### Quality Assurance of Data Collection

Four nutritionists served as data collectors in this study. They lived in each village during the data collection period, because access to the area was quite difficult. Data collection was carried out directly via face-to-face interviews through home visits by data collectors, who were accompanied by posyandu cadres in every village, with periodic supervision to ensure the accuracy of data collection. All instruments used in this study were calibrated before use. All data collectors had been given direction and were trained to be skilled in the use of these tools. Supervision was carried out by two practitioners, each with a master’s degree in public health nutrition as well as experience in research. The questionnaire used was reviewed by experts and tested previously on 10% of the total participants in other villages with similar characteristics to the study area. Once the data was collected, it was verified and checked for completeness by the data collector before being submitted to the supervisor.

#### Data processing and analysis

All items in the questionnaire were checked for missing values, including mother’s and child’s characteristics and environmental sanitation. Furthermore, it was coded and input in statistics software using SPSS version 22.0. Descriptive statistics consist of the mean, standard deviation, and percentage analysed by univariate analysis. Bivariate analysis uses the chi-square test, where the variables are categorical data. A 95% confidence level and a value of *P* < 0.05 are used to assess statistical significance. Binary logistic regression analysis are used to analyse the dominant factors associated with malnutrition based on underweight, stunting, wasting and the CIAF. Independent variables were entered in binary logistic regression analysis to determine the most dominant determinant associated with the dependent variables, namely, anthropometric failure (overall prevalence of undernutrition based on the CIAF) and others (underweight, stunting, wasting (measurement with a single or conventional anthropometric index), can be seen in Table 2. These independent variables are selected through candidate selection.

**Table 2:** Independent variables entered in binary logistic regression analysis based on dependent variable

Dependent Variable	Independent Variables
**A. Nutritional Status based on Composite Index of Anthropometric Failure (CIAF)**	
Anthropometric failure	Mother’s height, mother’s education, mother’s working status, child’s age, history of infectious diseases, early initiation of breastfeeding, frequency of consumption of energy sources, frequency of consumption of protein sources, cooking water source
**B. Nutritional Status based on Conventional (Single) Anthropometric Index**	
Underweight	Mother’s age, mother’s height, mother’s education, family income, child age, early initiation of breastfeeding, frequency of consumption of energy sources, frequency of consumption of protein sources, cooking water source, hand-washing habits
Stunting	Mother’s height, mother’s education, mother’s working status, family income, parenting, child’s age, history of infectious diseases, early initiation of breastfeeding, frequency of consumption of energy sources, frequency of consumption of protein sources, cooking water source
Wasting	Child’s age, history of infectious diseases, home’s relative humidity

## Results

Table 3 presented that the prevalence of anthropometric failure – that is, overall prevalence of undernutrition based on the CIAF – in children under five years old was 42.1%. This figure accounts for wasting only (2.4%), wasting and underweight (5.8%), wasting, underweight and stunting (2.1%), underweight and stunting (16.4%), stunting only (11.5%) and underweight only (3.9%). In contrast, the prevalence of other forms of undernutrition based on conventional anthropometrix indices, namely underweight, stunting and wasting were 27.8, 29.7 and 10.6%, respectively. Also, Table 3 illustrated that the percentage of children under five who experienced anthropometric failure, underweight and stunting among children aged 25–59 months was 53.8, 37.4 and 43.8%, respectively. This was higher than the corresponding values for children aged 0–24 months – that is, 29.6, 17.6 and 14.4%, respectively. The percentage of anthopometric failure (43.9%), underweight (29.3%) and stunting (32.4%) in boys is higher than in girls (40.4, 26.5, 27.1%, respectively). On the other hand, the proportion of children in the age range of 0–24 months who experience wasting (15.1%) is higher compared to children aged 25–59 months (6.4%); in addition, the proportion of girls who are wasting (11.4%) is higher than the proportion of boys (9.7%).

**Table 3:** Classification of nutritional status in children under five years old

Categories	Aged (0–24 months)		Aged (25–59 months)		Boys		Girls		Total	
	n	%	n	%	n	%	n	%	n	%
**Nutritional Status based on Composite Index of Anthropometric (Failure) CIAF)**										
No failure (A)	112	70.4	79	46.2	92	56.1	99	59.6	191	57.9
Wasting only (B)	8	5.0	0	0.0	5	3.0	3	1.8	8	2.4
Wasting & underweight (C)	13	8.2	6	3.5	7	4.3	12	7.2	19	5.8
Wasting, underweight & stunting (D)	2	1.3	5	2.9	4	2.4	3	1.8	7	2.1
Stunting and underweight (E)	12	7.5	42	24.6	30	18.3	24	14.6	54	16.4
Stunting only (F)	10	6.3	28	16.4	19	11.6	19	11.4	38	11.5
Underweight only (Y)	2	1.3	11	6.4	7	4.3	6	3.6	13	3.9
Anthropometric Failure (B + C + D + E + F + Y)	47	29.6	92	53.8	72	43.9	67	40.4	139	42.1
**Nutritional Status based on Conventional (Single) Anthropometric Index**										
**Weight-for-Age**										
Severely underweight (< −3 SD)	4	2.5	11	6.4	8	4.9	7	4.2	15	4.5
Underweight (− 3 SD to < −2 SD)	24	15.1	53	31.0	40	24.4	37	22.3	77	23.3
Normal weight (−2 SD to + 1 SD)	103	64.8	97	56.7	94	57.3	106	63.9	200	60.6
Risk of overweight (> + 1 SD)	28	17.6	10	5.9	22	13.4	16	9.6	38	11.6
**Length/Height-for-Age**										
Severely stunted (< −3 SD)	4	2.5	31	18.1	21	12.9	14	8.4	35	10.6
Stunted (−3 SD to < −2 SD)	19	11.9	44	25.7	32	19.5	31	18.7	63	19.1
Normal −2 SD to + 3 SD	130	81.8	96	56.2	107	65.2	119	71.7	226	68.5
Tall (> + 3 SD)	6	3.8	0	0.0	4	2.4	2	1.2	6	1.8
**Weight-for-length/height**										
Severely wasted (< −3 SD)	8	5.0	5	2.9	5	3.0	8	4.8	13	3.9
Wasted (−3 SD to < −2 SD)	16	10.1	6	3.5	11	6.7	11	6.6	22	6.7
Normal (−2 SD to + 1 SD)	119	74.9	147	86.0	129	78.8	137	82.6	266	80.6
Possible risk of overweight (> + 1 SD to + 2 SD)	8	5.0	10	5.8	11	6.7	7	4.2	18	5.5
Overweight (> + 2 SD to + 3 SD)	3	1.9	0	0.0	3	1.8	0	0.0	3	0.9
Obese (> + 3 SD)	5	3.1	3	1.8	5	3.0	3	1.8	8	2.4

Table 4 shown that the percentage (80.4%) of anthropometric failure (overall prevalence of undernutrition based on the CIAF) is higher for mothers of 25 years or older. Similarly, the percentage for underweight (75.5%), stunting (82.9%) and wasting (77.7%) in children under five is higher for mothers of 25 years or older than for mothers younger than 25. The percentage of children under five who experienced anthropometric failure (57.6%), underweight (59.8%) and stunting (64.3%) was higher for short mothers (height below 150 cm) than tall mothers (height is equal to or more than 150 cm). Meanwhile, the percentage of wasting children was higher for tall mothers (57.1%) than for short mothers (42.9%). The percentage of children under five who suffer anthropometric failure (71.2%), underweight (71.7%), stunting (75.5%) or wasting (65.7%) was higher for mothers with low education than for mothers with higher education. The percentage of children under five suffering from anthropometric failure (77.0%), underweight (78.3%), stunting (71.4%) or wasting (85.7%) was higher for mothers who did not work (housewives) than for employed mothers. The percentage of children under five suffering from anthropometric failure (55.6%), underweight (70.6%), stunting (56.0%) or wasting (60.0%) was higher in families with low income levels (below the regional minimum wage) than for families with high income levels. The percentage of children under five who experienced anthropometric failure (66.9%), underweight (67.4%), stunting (66.3%) or wasting (71.4%) was higher for mothers who were grand multiparity than for mothers who were prirmiparity and grand multiparity. The percentage of children under five who experienced anthropometric failure (74.1%), underweight (73.9%), stunting (73.5%) or wasting (80.0%) was higher for mothers with low nutritional knowledge than for mothers with high nutritional knowledge. The percentage of children under five who experienced anthropometric failure (51.8%), underweight (52.2%) or stunting (54.1%) was higher in boys than girls. However, under five wasting was more prevalent for girls (54.3%) than for boys (45.7%). The percentage of children under five who experienced anthropometric failure (66.2%), underweight (69.6%) or stunting (76.5%) was higher among children aged 25–59 months than among children aged 0–24 months. On the other hand, the percentage of wasting toddlers was higher in toddlers aged 0–24 months (68.6%) than toddlers aged 25–59 months (31.4%). In this study, it can be seen that the percentage of children under five who experienced anthropometric failure (66.2%), underweight (64.1%), stunting (66.3%) or wasting (71.4%) was higher among children under five who had a history of infectious diseases than among children under five who had no history of infectious disease. The percentage of children under five who experienced the four nutritional problems was also more common among children under five who were not fully immunized, i.e. 49.6, 50.0, 49.0 and 48.6%, than children under five who were completely immunized.

**Table 4:** Maternal and child characteristic based on anthropometric failure (overall prevalence of undernutrition using CIAF) and other undernutrition (*n* = 330)

Characteristics	Anthropometric Failure using the CIAF n (%)	Conventional Anthropometric index		
		Underweight n (%)	Stunting n (%)	Wasting n (%)
**Maternal Characteristics**				
**Age**				
< 25 years	31 (22.3)	18 (19.6)	24 (24.5)	6 (71.1)
≥ 25 years	108 (77.7)	74 (80.4)	74 (75.5)	29 (82.9)
**Height**				
Short (< 150 cm)	80 (57.6)	55 (59.8)	63 (64.3)	15 (42.9)
Tall (≥ 150 cm)	59 (42.4)	37 (40.2)	35 (35.7)	20 (57.1)
**Education**				
Low	99 (71.2)	66 (71.7)	74 (75.5)	23 (65.7)
High	40 (28.8)	26 (28.3)	24 (24.5)	12 (34.3)
**Working Status**				
Unemployed	107 (77.0)	72 (78.3)	70 (71.4)	30 (85.7)
Employed	32 (23.0)	20 (21.7)	28 (28.6)	5 (14.3)
**Family Income**				
Low (below the regional minimum wage)	15 (55.6)	14 (56.0)	3 (60.0)	15 (55.6)
High (equal or more than the regional minimum wage)	12 (44.4)	11 (44.0)	2 (40.0)	12 (44.4)
**Parity**				
Primiparity	28 (20.1)	19 (20.7)	20 (20.4)	7 (20.0)
Multiparity	93 (66.9)	62 (67.4)	65 (66.3)	25 (71.4)
Grand multiparity	18 (12.9)	11 (12.0)	13 (13.3)	3 (8.6)
**Nutrition knowledge**				
Not good	103 (74.1)	68 (73.9)	72 (73.5)	28 (80.0)
Good	36 (25.9)	24 (26.1)	26 (26.5)	7 (20.0)
**Parenting**				
Other	16 (11.5)	11 (12.0)	15 (15.3)	2 (5.7)
Mother	123 (88.5)	81 (88.0)	83 (4.7)	33 (94.3)
**Child Characteristics**				
**Sex**				
Male	72 (51.8)	48 (52.2)	53 (54.1)	16 (45.7)
Female	67 (48.2)	44 (47.8)	45 (45.9)	19 (54.3)
**Age**				
0–24 months	47 (33.8)	28 (30.4)	23 (35.5)	24 (68.6)
25–59 months	92 (66.2)	64 (69.6)	75 (76.5)	11 (31.4)
**History of Infectious Diseases**				
Yes	92 (66.2)	59 (64.1)	65 (66.3)	25 (71.4)
No	47 (33.8)	33 (35.9)	33 (33.7)	10 (28.6)
**History of Immunization**				
Ungiven	14 (10.1)	8 (8.7)	7 (7.1)	5 (14.3)
Incomplete	69 (49.6)	46 (50.0)	48 (49.0)	17 (48.6)
Complete	56 (40.3)	38 (41.3)	43 (43.9)	13 (37.1)
**Early Initiation of Breastfeeding**				
No	101 (72.7)	66 (71.7)	73 (74.5)	25 (71.4)
Yes	38 (27.3)	26 (28.3)	25 (25.5)	10 (28.6)
**Frequency consumption of energy sources**				
Low (< 3x/day)	10 (7.4)	7 (7.8)	2 (2.1)	3 (9.1)
High (≥ 3x/day)	125 (92.6)	83 (92.2)	94 (97.9)	30 (90.9)
**Frequency consumption of protein sources**				
Low (< 3x/day)	27 (20.6)	15 (17.2)	16 (17.2)	10 (31.3)
High (≥ 3x/day)	104 (79.4)	72 (82.8)	77 (82.8)	22 (68.8)
**Environment sanitation and clean living behaviour**				
**Ambient room temperature**				
Uncomfortable (< 20.5 °C and > 27.2 °C)	62 (78.5)	44 (81.5)	46 (79.3)	12 (75.0
Comfortable (20.5 °C–27.2 °C)	17 (21.5)	10 (18.5)	12 (20.7)	4 (25.0)
**Home’s Relative Humidity**				
Uncomfortable (< 40% and > 70%)	65 (86.7)	41 (82.0)	46 (85.2)	16 (100.0)
Comfortable (40–70%)	10 (13.3)	9 (18.0)	8 (14.8)	0 (0.0)
**Cooking water source**				
Unprotected springs	5 (3.6)	4 (4.3)	4 (4.1)	0 (0.0)
Protected springs	134 (96.4)	88 (95.7)	94 (95.9)	35 (100.0)
**Handwashing habit**				
Not good	70 (50.4)	43 (46.7)	53 (54.1)	16 (45.7)
Good	69 (49.6)	49 (53.3)	45 (45.9)	19 (54.3)
**Defecation habit**				
Open place	3 (2.2)	3 (3.3)	2 (2.0)	0 (0.0)
Toilet	136 (97.8)	89 (96.7)	96 (98.0)	35 (100.0)

In Table 5**,** it can also be seen that the percentage of children under five who experienced anthropometric failure (72.7%), underweight (71.7%), stunting (74.5%) or wasting (71.4%) was higher among children who were not given EIB than among children under five who were given EIB. In this study, it is also known that children under five who experience anthropometric failure and other types of undernutrition are higher in children under five who consume protein and energy sources of food equal or more than three times a day compared to children under five who consume protein and energy sources below three times a day. In addition, the percentage of children under five who experienced anthropometric failure (78.5%), underweight (81.5%), stunting (79.3%) or wasting (75.0%) was higher for children under five who lived at home with uncomfortable ambient room temperature (< 20.5 °C and > 27.2 °C) than for children under five who lived in a house with a comfortable ambient room temperature (20.5 °C–27.2 °C). Likewise, the percentage of toddlers who experience anthropometric failure (86.7%), underweight (82.0%), stunting (85.2%) or wasting (100%) is also higher for children under five who live at home with uncomfortable relative humadity (< 40% or > 70%) than children under five who live in a house with comfortable relative humidity (40–70%). Almost all children under five (95–100%) who experienced undernutrition in this study came from families that used cooking water sources from protected springs. The percentage of children under five who experience anthropometric failure is not significantly different between children under five who lack good hand-washing habits (50.4%) and children under five who have good hand washing-habits (49.6%). Children under five who experience anthropometric failure and other undernutrition (underweight, stunting and wasting) are almost all in the habit of defecating in the toilet. Table 5 shown that, based on bivariate analysis, there are factors associated with anthropometric failure (overall prevalence of undernutrition based on the CIAF), underweight, stunting and wasting. The results of this study indicate that there are four factors associated with the occurrence of underweright in children under five years, namely, mother’s height (*p* = 0.028), family income (*p* = 0.043), child’s age (*p* < 0.001) and frequency of consumption of protein sources (*p* = 0.015). Meanwhile, other factors showed no significant relationship (*p* > 0.05) with underweight in children under five. There are eight variables related to stunting, including mother’s height (*p* = 0.001), mother’s education (*p* = 0.018), mother’s occupation (*p* = 0.013), parenting (*p* = 0.029), child’s age (*p* < 0.001), EIB (*p* = 0.034), frequency of consumption of energy sources (*p* = 0.001) and protein (*p* = 0.010). Other factors were not significantly related (*p* > 0.05) with the incidence of stunting in children under five. In this study, it is also known that there is only one variable that is significantly related to wasting, namely, the child’s age (*p* = 0.018). Other variables showed no significant relationship (*p* > 0.05) on the incidence of wasting. There are six factors that are significantly related to the occurrence of anthropometric failure, namely, mother’s height (*p* = 0.017), child’s age (*p* < 0.001), history of infectious disease (*p* = 0.034), EIB (*p* = 0.026), frequency of consumption of energy sources (*p* = 0.047) and frequency of consumption of protein sources (*p* = 0.024). Other factors showed no significant relationship (*p* > 0.05) to anthropometric failure.

**Table 5:** Factors associated with undernutrition (underweight, stunting, wasting and anthropometric failure) in children under five years old

Characteristics	Underweight		Stunting		Wasting		Anthropometric Failure using the CIAF	
	***P-value***	OR (95% CI)	***P-value***	OR (95% CI)	***P-value***	OR (95% CI)	***P-value***	OR (95% CI)
**A. Mother Characteristics**								
Age	0.189	0.64 (0.35–1.16)	0.967	0.95 (0.55–1.64)	0.343	0.59 (0.23–1.46)	0.374	0.77 (0.46–1.28)
Height	**0.028**	1.78 (1.09–2.90)	**0.001**	2.36 (1.45–3.85)	0.510	0.74 (0.36–1.50)	**0.017**	1.75 (1.13–2.73)
Education	0.173	1.49 (0.88–2.52)	**0.018**	1.65 (1.15–3.32)	1.000	1.01 (0.48–2.12)	0.078	1.56 (0.98–2.50)
Working Status	0.670	0.84 (0.46–1.52)	**0.013**	0.47 (0.27–0.83)	0.531	1.53 (0.57–4.12)	0.248	0.70 (0.40–1.20)
Family Income	**0.043**	4.11 (1.19–14.13)	0.362	1.91 (0.65–5.60)	0.898	1.76 (0.27–11.47)	0.348	1.93 (0.66–5.65)
Parity	0.617	–	0.620	–	0.551	–	0.379	–
Nutrition knowledge	0.871	1.09 (0.63–1.87)	0.951	1.05 (0.62–1.80)	0.412	1.57 (0.66–3.72)	0.724	1.13 (0.67–1.85)
Parenting	0.434	1.48 (0.68–3.22)	**0.029**	2.44 (1.15–5.15)	0.629	0.56 (0.13–2.44)	0.351	1.53 (0.73–3.20)
**B. Child Characteristics**								
Sex	0.662	1.15 (0.71–1.86)	0.360	1.28 (0.80–2.06)	0.749	0.84 (0.41–1.69)	0.589	1.16 (0.75–1.80)
Age	**< 0.001**	0.36 (0.21–0.60)	**< 0.001**	0.22 (0.13–0.37)	**0.018**	2.59 (1.22–5.47)	**< 0.001**	0.36 (0.23–0.57)
History of Infectious Diseases	0.302	1.34 (0.81–2.20)	0.106	1.54 (0.94–2.53)	0.165	1.84 (0.85–3.97)	**0.034**	1.67 (106–2.63)
History of Immunization	0.821	–	0.450	–	0.426	–	0.526	–
Early Initiation of Breastfeeding	0.173	0.67 (0.40–1.13)	**0.034**	0.55 (0.32–0.93)	0.550	0.73 (0.34–1.59)	**0.026**	0.57 (0.35–0.91)
Frequency consumption of energy sources	0.218	0.53 (0.22–1.28)	**0.001**	0.11 (0.02–0.45)	0.820	0.72 (0.21–2.51)	**0.047**	0.43 (0.20–0.94)
Frequency consumption of protein sources	**0.015**	0.44 (0.24–0.83)	**0.010**	0.43 (0.23–0.80)	0.765	1.23 (0.55–2.73)	**0.024**	0.52 (0.30–0.90)
**Environment Sanitation and Clean Living Behaviour**								
Room temperature	0.618	1.32 (0.60–2.93)	0.955	1.10 (0.52–2.34)	0.477	0.82 (0.25–2.70)	1.000	1.03 (0.51–2.08)
Humidity	0.284	0.54 (0.22–1.35)	0.756	0.77 (0.31–1.94)	0.103	–	1.000	0.91 (0.38–2.20)
Cooking water source	0.098	3.56 (0.78–16.23)	0.120	3.25 (0.71–14.80)	0.764	–	0.230	3.53 (0.67–18.45)
Hand washing habit	0.218	0.71 (0.44–1.16)	0.842	1.08 (0.67–1.73)	0.484	0.73 (0.36–1.47)	0.533	0.85 (0.55–1.31)
Defecation habit	0.394	1.57 (0.37–6.71)	0.559	0.78 (0.16–3.95)	0.404	–	0.545	0.82 (0.19–3.50)

Table 6 presented are binary logistic regression analysis shows that family income is the dominant factor influencing anthropometric failure (overall prevalence of undernutrition using the CIAF) is mother’s height (*p* = 0.008, AOR = 1.95, 95% CI: 1.19–3.19). From these results, it can be concluded that short mothers are 1.95 times more likely that tall mothers to have children who experience anthropometric failure. In addition, anthropometric failure is also influenced by the child’s age (*p* = 0.046, AOR = 0.57, 95% CI: 0.32–1.00). The dominant factor associated with underweight is family income (*p* = 0.018, OR: 5.44, 95% CI: 1.34–22.11). It can be concluded that families with low income levels are 5.4 times more likely to have underweight children than families with high income. Another factor that causes underweight is a child’s age (*p* = 0.026, AOR = 0.07, 95% CI: 0.006–0.72). The dominant factors associated with stunting are mother’s height (*p* < 0.001; AOR: 3.29, 95% CI: 1.83–5.91). From this study, it can be seen that short mothers are at 3.29 times higher risk than tall mothers of having children who experience stunting. Moreover, a child’s age (*p* = 0.002, AOR = 0.34, 95% CI: 0.17–0.66) and the frequency of consumption of energy sources (*p* = 0.003, AOR = 0.09, 95% CI: 0.02–0.46) are also associated with stunting. The dominant factor related to wasting is a child’s age child (*p* = 0.013, AOR: 2.59, 95% CI: 1.22–5.47). Children aged 25–59 months had a 2.4 higher risk of suffering from wasting than children aged 0–24 months.

**Table 6:** Binary logistic regression analysis of factors associated with undernutrition (CIAF, underweight, stunting and wasting) in children under five years old

Categories	Characteristics	p-value	Adjusted OR	95% CI
**Composite Index of Anthropometric Failure (CIAF)**				
Anthropometric failure	Mother’s height	0.008	1.95	1.19–3.19
	Child’s age	0.046	0.57	0.32–1.00
**Conventional Anthropometric Index**				
Underweight	Family income	0.018	5.44	1.34–22.11
	Child’s age	0.026	0.07	0.006–0.72
Stunting	Mother’s height	< 0.001	3.29	1.83–5.91
	Child’s age	0.002	0.34	0.17–0.66
	Frequency consumption of energy sources	0.003	0.09	0.02–0.46
Wasting	Child’s age	0.013	2.59	1.22–5.47

## Discussion

### Overview of anthropometric failure and other undernutrition in children under five

In this study, it can be seen that nearly half (42.1%) of children under five years of age experience anthropometric failure (overall prevalence of undernutrition using the CIAF), consisting of wasting only (2.4%), wasting and underweight (5.8%), wasting, underweight and stunting (2.1%), underweight and stunting (16.4%), stunting only (11.5%) and underweight only (3.9%). Meanwhile, based on conventional anthropometric indices, almost one third of children under five are underweight (27.8%), one third are stunting (29.7%) and about one tenth (10.6%) of toddlers experience wasting.

In general, these studies report that the high prevalence of anthropometric failure using the CIAF and various other kinds of undernutrition as measured by conventional anthropometric indices is associated with sociodemographic characteristics of the population in rural areas, as well as characteristics pertaining to food intake, environmental sanitation conditions and clean living behaviour [25, 44, 45]. Islam and Biswas in Bangladesh reported a higher prevalence of toddlers experiencing anthropometric failure than that indicated by this study, which was almost 49%. Of this figure, 18.2% is comprised of a combination of stunting and underweight, 5.5% is due to combination of wasting and underweight, and 5.7% is from a combination of wasting, underweight and stunting.

The study also found that most of the anthropometric failures occurred in children under five who live in rural areas and fall into the category of poor people. In addition, the prevalence of anthropometric failure is higher among infants with a higher birth order and not receiving vaccinations. The high prevalence of anthropometric failure in various studies is associated with the goal of measuring nutritional status using the CIAF. More specifically, this measurement shows the overall prevalence of malnutrition, which provides six mutually exclusive anthropometric measurements, including length/height-for-age, length/height-for-weight and weight-for-age [46]. Undernutrition as measured by conventional anthropometric indices shows a similar prevalence as regards underweight, stunting and wasting. The three forms of undernutrition associated with sociodemographic factors were also significantly associated with low food intake, poor environmental sanitation conditions and poor clean living practices [25, 44, 45].

### Maternal and child characteristics

Based on this study, it can be seen that the participants are people who live in rural areas with low socioeconomic levels. The majority of the participating mothers are housewives who are the main caregivers for children under five. These mothers also have low levels of education, low levels of family income, more than two children and poor nutritional knowledge; the majority also have short stature. Nutritional problems in rural areas are associated with food security problems, i.e. the low availability of food and the ability of the community to access it [46]. In this study, it can also be seen that anthropometric failure and other undernutrition is higher among boys than among girls. In addition, undernutrition is more common in children aged 25–59 months compared to those aged 0–24 months. This is associated with the greater need for calories and physical activity among boys, compared to girls. In addition, as the baby grows, the nutritional needs will be higher and their physical activity will increase [47].

Other findings in this study also show that undernutrition in children under five was also higher in prevalence for infants who were given incomplete immunizations, had a history of suffering from infectious diseases or were not given EIB. Exclusive breastfeeding from the time the baby is born to the first six months of life, accompanied by complete immunization, can increase the baby’s immunity so that it can prevent the occurrence of various infectious diseases, such as Acute Resporatory Infection (ARI). Breast milk contains various antibodies that can significantly increase the expression of Natural Resistance-Associated Macrophage Protein 1 (NRAMP1), gene mRNA and NRAMP1 protein levels to form body immunity [18]. It also contains Secretory Immunoglobulin A (sIgA) and lactoferrin, which also play a role in preventing the occurrence of infectious diseases such as ARI [48]. This study shows that most of the children had consumed food sources of energy and food sources of protein with a frequency equal to or greater than three times a day. Providing the right frequency of consumption of energy and protein sources, the appropriate amount of the Recommended Dietary Allowance (RDA), as well as varying the types, can prevent undernutrition in children under five [49].

### Environment sanitation and clean living behaviour

In this study, environmental sanitation variables were not associated with undernutrition based on anthropometric failure (overall prevalence using the CIAF), underweight, stunting or wasting. However, this study shows that under-five children who experience undernutrition tend to be more common among those who live in environments with poor sanitation and home thermal comfort than children under five who live in good sanitation environments and home thermal comfort. Studies in Indonesia show that climate change, which causes a crisis in food availability and exposure to environmental temperatures including room temperature and relative humdaity, has a significant impact on the occurrence of undernutrition in children under five [33].

Tusting et al. (2020) found that children living in hotter regions of sub-Saharan Africa were more likely to be wasting, underweight and simultaneously wasting and stunting, but were less likely to be stunting than those in colder regions [50]. Research conducted in the same location in 2019 showed that an increase in temperature exposure for 470 hours above 30 °C raised the possibility of an increase in wasted by 3% and stunting by 6% [51]. Research conducted in an urban area of Bangladesh found that environmental sanitation is associated with undernutrition in children [52]. Studies in Brazil have shown that there is an increase in patients suffering from malnutrition in summer: Every 1 °C increase in average daily temperature is associated with a 2.5% increase in hospitalizations due to undernutrition for up to seven days [53].

Uncomfortable home temperatures, particularly in hot temperatures, are associated with disturbances to children’s health. This is because, although the body has the ability to respond to environmental heat and adapt to it, exposure to extreme heat can overcome the human body’s resistance and can increase the risk of disease that has an impact on nutritional status. Likewise, uncomfortable relative humidity at home is also associated with an increased risk of various infectious diseases [54]. In this study, clean living behaviour is not related to undernutrition based on conventional anthropometric index (underweight, stunting, wasting) or anthropometric failure (total prevalence of undernutrition using the CIAF). This study is inconsistent with an observational study in rural Bangladesh finding that environmental pollution associated with open defecation causes linear growth retardation through environmental enteropathy – and that children in clean household environments have higher WAZ scores, by 0.54 standard deviations, than children living in dirty environments [55]. Washing hands with good soap can prevent almost half of all cases of diarrhea in children. Another study showed that the availability of clean water and the consumption of drinking water contaminated with coliform bacteria were positively related to the occurrence of diarrhea and undernutrition in children under five [34]. Drinking clean water and washing one’s hands with soap are also thought to enhance nutrition by preventing diarrhea and reducing stunting by up to 15% for children under five years, providing them with a better chance of maintaining good health and growing up to thrive [20].

### Nutritional status based on conventional anthropometric index and influencing factors

#### Underweight (weight-for-age) and influencing factors

This study found family income to be the most dominant factor associated with underweight in children under five. Families with low income levels are 5.44 times more likely to have underweight children than families with high incomes. Globally, it is reported that substantial increases in mortality and overall disease burden are due to child malnutrition in low-income and middle-income countries [56]. A study conducted by Chowdhury et al. in Bangladesh showed that underweight in children under five are associated with household position in lower wealth index. Children under five who come from families with low income levels also have uneducated mothers; this factor is one of the determinants of underweight [57].

Low family income is related to low parental education, lack of nutritional knowledge and low paying jobs. It is also associated with household food insecurity, where families experience limitations in physical and economic access to safe, sufficient and nutritious food to meet their food needs and food preferences and to achieve a productive, healthy and active life. In addition, low family income is related to family limitations in accessing better health care facilities [58]. Another dominant factor related to underweight, in this study, is the age of the child. Toddlers aged 25–59 months are at a greater risk of being underweight compared to toddlers aged 0–24 months. This result is in line with a study conducted by Sinha et al. in five high-burden pockets of four Indian states, which shows that age is significantly associated with the occurrence of underweight; older children (25–59 months) are at a higher risk of being underweight than younger children (0–24 months). This is associated with changes in diet and physical activity. At the age of more than 24 months, toddlers have also stopped breastfeeding, so they must fully meet the macronutrient and micronutrient needs from food intake. Meanwhile, there is an increase in physical activity in older children. Imbalance between food intake and physical activity also causes undernutrition in toddlers [59].

#### Stunting (length/height-for-age) and influencing factors

Based on the results of binary logistic regression analysis in this study, it is known that the most dominant factor associated with stunting is the mother’s height. Short mothers (height below 150 cm) have a 3.29 times greater risk of having stunting toddlers than tall mothers (height equal or more than 150 cm). According to WHO, a 19-year-old woman with a height of less than − 2 standard deviations (less than 150 cm) has a short stature [60]. This result is in line with a study conducted by Ali et al. in Northern Ghana, which showed that short mothers (less than 150 cm in height) were 3.87 times more likely to have stunted children than tall mothers. That study also reported that an increase in maternal height of 1 cm could reduce the risk of stunting by 1%. this shows that there is an intergenerational transmission of stunting, where shorter mothers tend to have stunted children, then the child in the next period of life will become a short mother and have stunting children. In addition, short mothers who have experienced undernutrition in the past may experience macronutrient and micronutrient deficiencies during pregnancy and lactation, so they are at risk of having stunted children [61].

Another dominant factor associated with stunting in children under five in this study is the child’s age. A similar study by Sinha et al. in five high-burden pockets of four Indian states shows that a child’s age is a strong determinant of stunting. Older children aged 24–59 months are at a higher risk of stunting than younger children aged 0–24 months Stunting occurs when toddlers are malnourished during the first 1000 days of life. This stunting begins to appear when the baby is two years old; at this age, the child experiences a transition period, namely, from the period of breastfeeding to switching to food intake as the sole means for meeting its nutritional needs. Inappropriate feeding at this time leads to unfulfilled nutritional needs and causes growth failure [59]. Another finding from this study shows that a further dominant factor associated with stunting is the frequency of consumption of energy sources. Children under five who have a low frequency of consumption of energy source foods (below three times a day) are at a greater risk of stunting than children under five who have a high frequency of consumption of energy source foods. This finding aligns with a study by Laurus, Fatimah and Gurnida in Jatinangor, West Java province, Indonesia, which reported that stunting children have a low frequency of consumption of energy source foods (less than three times per day), with the most frequently consumed types of food being white rice and biscuits and the number of calories being below the RDA. The fulfillment of energy needs contributes to achieving optimal growth and can prevent stunting [62].

#### Wasting (weight-for-height) and influencing factors

The dominant factor associated with wasting is the child’s age. The prevalence of wasting is higher among children aged 0–24 months than among children aged 25–59 months. Scientific evidence suggests that wasting occurs mainly during the first two years of life. A study conducted by Wali, Agho and Renzaho in five countries in South Asia showed that age is strongly associated with wasting in toddlers; the prevalence of wasting was higher in children aged 0–23 months compared to children aged 24–59 months at 25 and 18%, respectively [63]. Another study conducted on wasting children in Bhutan showed that the prevalence of underweight children was more than twice as high among children aged 0–23 months (9.2%) than among children aged 24–59 months (3.8%), while the prevalence of severe wasting was almost four times higher among children aged 0–23 months (3.8%) than among children aged 24–59 months (1.0%) [64].

A study by Karlsson et al. in 94 mostly low and middle-income countries shows that the prevalence of wasting was higher for toddlers aged 0–23 months (14%) than for toddlers older than 24 months (9%). The occurrence of wasting is associated with the fulfillment of food intake and the occurrence of infectious diseases during the first two years of a child’s life. If the child lacks food intake, then weight loss occurs more quickly, so the child becomes thin. In addition, the immune system also matures according to the age of the child. At the age of 0–24 months, children are at a greater risk of getting infectious diseases, such as diarrhea which have an impact on weight loss; thus, they suffer from wasting [65].

#### Anthropometric failure using the CIAF and influencing factors

In this study, the most dominant factor associated with anthropometric failure in children under five is the mother’s height. Short mothers have a 1.95 times higher risk of having children who experience anthropometric failure, compared to tall mothers. This aligns with the study conducted by Addo et al., which found that a mother’s height is the strongest factor that causes growth failure. That study reported that a 1 cm increase in maternal height predicted a 0.024 (95% CI: 0.021–0.028) SD increase in offspring birth weight and a 0.037 (95% CI: 0.033–0.040) SD increase in conditional height at two years of age.

The study also reported that mothers with a height below 150 cm were more likely to have children who experienced anthropometric failure at the age of two years, with a prevalence ratio of 3.20 [66]. The study contends that the relationship between maternal height and child growth is a condition that occurs as the outcome of the interaction between genetic and environmental factors [25]. Height is one of the most heritable quantitative phenotypes of humans. Genetic effects are associated with genome-wide association studies (GWA) that allow the identification of sites consistently associated with height in populations from different ancestors [67].

In addition, short mothers indicate chronic malnutrition in the period before pregnancy; this occurs as a result of low social and economic conditions, especially for those who live in rural areas. Short mothers tend to have small reproductive organs, so they have a narrower space for the fetus. The higher the age of the fetus in the womb, the greater the need for protein and energy. If the size of the uterus is small, then the storage for protein and energy becomes limited. If a short mother suffers undernutrition during the pregnancy period, the fetal growth becomes sub-optimal. They are at risk of giving birth to small babies who experience growth that is not in accordance with their gestational age, which in turn carries a higher risk of malnutrition [25].

Another dominant factor related to anthropometric failure in this study is the child’s age. Children aged 25–59 months are at a higher risk of experiencing anthropometric failure, compared to toddlers aged 0–24 months. Various studies show results that are in line with this finding [29, 30, 44, 46, 68–70].. This result is also in line with EIB in the 2011 Ethiopian Demographic and Health Surveys (EDHS) survey [31]. This study shows that children in the older age group had a higher risk of undernutrition than the younger age group. Children at a younger age get more optimal nutritional intake, i.e. from breastfeeding to weaning. However, as children get older, they need more nutrition, which must be provided by their daily intake. In addition, older toddlers have higher levels of physical activity, so they need higher levels of food intake than younger children [31].

## Conclusions

Anthropometric failure (overall prevalence of undernutrition based on the CIAF) occurred in almost half of the children under five in this study. This measurement describes the total of all types of undernutrition, so as to identify undernutrition comprehensively. The dominant determinant that causes anthropometric failure in this study is the mother’s height; short mothers are 1.95 times more likely than tall mothers to have children who experience anthropometric failures. The results of this study strengthen scientific evidence that the measurement of nutritional status using the CIAF can comprehensively identify the overall undernutrition in children under five. The results of this study can also serve as a basis for determining appropriate interventions to prevent early undernutrition, so that the prevalence of undernutrition in children under five can be reduced. There is a need for sensitive multi-sectoral nutrition interventions and specific nutrition interventions to improve the nutrition of prospective mothers, which is carried out from the pre-pregnancy period, especially during the second stage of rapid growth during adolescence. Similarly, the nutrition improvement for children during the first 1000 days of life is needed to determine optimal nutritional status as an indicator of growth success.

### Limitations of the study

This study only uses one sub-district (consisting of two community health centres) out of 40 sub-districts in Bogor Regency. The area was used as a research location because it has the highest prevalence of stunting in Bogor Regency, based on data from the Bogor Regency Health Office. However, it is necessary to add research locations from several other sub-districts, in order to obtain generalizable research results. In addition, not all participants’ residences were measured for room temperature and humidity, so it did not fully describe the condition of the two measurements for all of the participant’s residences. In order to provide better results and stronger evidence regarding the role of these two variables on the occurrence of under nutrition in children under five, it is necessary to measure all participants’ residences.

## Supplementary Information

**Additional file 1.**

## Data Availability

The datasets used and/or analysed during the current study are available from the corresponding author on reasonable request.

## Abbreviations

ARI: Acute Respiratory Infections
BMI: Body Mass Index
CIAF: Composite Index of Anthropometric Failure
COVID-19: Coronavirus Disease 2019
CSA: Central Statistics Agency
EDHS: Ethiopian Demographic and Health Surveys
EIB: Early Initiation of Breastfeeding
HAZ: Height for Age Z Score
NRAMP1: Natural Resistance-Associated Macrophage Protein 1
RDA: Recommended Dietary Allowance
SDGs: Sustainable Development Goals
SIGA: Secretory Immunoglobulin A
WAZ: Weight for Age Z Score
WHO: World Health Organization
WHZ: Weight for Height Z Score
