# Supplementary material for: Assessment of undernutrition using the composite index of anthropometric failure (CIAF) and its determinants: A cross-sectional study in the rural area of the Bogor District in Indonesia
Source: BMC Nutr. 2022 Nov 16;8:133. doi: 10.1186/s40795-022-00627-3 (PMC9666932; doi:10.1186/s40795-022-00627-3)
Supplement: Supplementary file 1 — Additional file 1. [file 40795_2022_627_MOESM1_ESM.docx]

**File name : Supplementary file 1**

**Title of data : Nutritional knowledge questionnaire**

**Description of data :** The nutritional knowledge questionnaire consists of 20 items. Mothers were asked to answer “true” or “false” for each statement. Each correct answer was given a score of ‘1’; each incorrect answer was given a score of ‘0’. The total score obtained is divided by the number of questions multiplied by 100 percent (correct answer score/20 x 100 percent).

**Instruction:**

Kindly fill in each of the following statements in Table 1 (Nutritional Knowledge Questionnaire) by putting a cross (x) in the answer column by choosing ‘**TRUE**’ or ‘**FALSE**’ answers according to the assessment that you think is the most appropriate.

| **No** | **STATEMENT/QUESTIONS** | **ANSWER** | |
| --- | --- | --- | --- |
|  |  | **TRUE** | **FALSE** |
|  | Balanced nutrition is a daily food composition that contains nutrients in the type and amount of the growing needs of children under five |  |  |
|  | Four principles of balanced nutrition are diverse foods consumption, clean and healthy living behavior, physical activity, and weighing regularly. |  |  |
|  | Consumption of a diverse and nutritionally balanced diet can support optimal growth in toddlers |  |  |
|  | My plate for toddlers consists of 35% staple foods, 35% side dishes, and 30% vegetables and fruit. |  |  |
|  | Rice, noodles, potatoes, cassava, and sweet potatoes are food sources of carbohydrates |  |  |
|  | Meat, eggs, fish, milk, and fish (example: tilapia, catfish, anchovies, cork, catfish) are food sources of animal protein |  |  |
|  | Tempe, tofu, and beans (example: peanuts, peas, kidney beans, soybeans) are food sources of vegetable protein |  |  |
|  | Cheese, butter, egg yolks, and oil are sources of fat |  |  |
|  | Vegetables and fruits are high sources of vitamins and minerals |  |  |
|  | Macronutrients are foods that the body needs in large quantities, consisting of carbohydrates, fats, and proteins |  |  |
|  | Micronutrients are foods that the body needs in small amounts, consisting of vitamins and minerals |  |  |
|  | Exclusive breastfeeding, namely giving only breast milk without adding any food and drinks except vitamins and drugs from the time the baby is born to the age of 6 months, is the best food for toddler’s growth. |  |  |
|  | Complementary foods such as porridge, bananas, biscuits, starch water, and fruit juices are given right from the time the baby is born |  |  |
|  | Complementary foods such as porridge, bananas, biscuits, starch water, and fruit juices are given right from the time the baby is born to gain weight |  |  |
|  | The weighing of the toddler is done at least every six months |  |  |
|  | Washing hands is enough to do after giving food to toddlers |  |  |
|  | Physical activities such as running, cycling, and playing can inhibit toddler's growth |  |  |
|  | Giving complete immunization to toddlers can prevent infectious diseases such as influenza, diarrhea, acute respiratory infection (ARI), and tuberculosis |  |  |
|  | Undernutrition in toddlers can cause growth and development disorders. |  |  |
|  | Stunting is a condition of malnutrition that occurs for a long time, where toddlers have a shorter height than children their age |  |  |
